# Associations of sensory impairment and cognitive function in middle-aged and older Chinese population: The China Health and Retirement Longitudinal Study

**DOI:** 10.7189/jogh.11.08008

**Published:** 2021-12-18

**Authors:** Xiaohuan Zhao, Yifan Zhou, Kunchen Wei, Xinyue Bai, Jingfa Zhang, Minwen Zhou, Xiaodong Sun

**Affiliations:** 1 Department of Ophthalmology, Shanghai General Hospital (Shanghai First People’s Hospital), Shanghai Jiao Tong University School of Medicine, Shanghai, China; 2 National Clinical Research Center for Eye Diseases, Shanghai, China; 3 Shanghai Key Laboratory of Fundus Diseases, Shanghai, China; 4 Putuo People’s Hospital, Tongji University, Shanghai 200060, China; 5 Department of Plastic and Reconstructive Surgery, Shanghai Ninth People's Hospital, Shanghai Jiaotong University School of Medicine, Shanghai, China

## Abstract

**Background:**

Little is known about the associations between vision impairment, hearing impairment, and cognitive function. The aim of this study was to examine whether vision and hearing impairment were associated with a high risk for cognitive impairment in middle-aged and older Chinese adults.

**Methods:**

A total of 13 914 Chinese adults from the China Health and Retirement Longitudinal Study (CHARLS) baseline were selected for analysis. Sensory impairment was assessed from a single self-report question, and we categorized sensory impairment into four groups: no sensory impairment, vision impairment, hearing impairment, and dual sensory impairment. Cognitive assessment covered memory, mental state, and cognition, and the data was obtained through a questionnaire.

**Results:**

Memory was negatively associated with hearing impairment (β = -0.043, 95% confidence interval (CI) = -0.076, -0.043) and dual sensory impairment (β = -0.033, 95% CI = -0.049, -0.017); mental status was negatively associated with vision impairment (β = -0.034, 95% CI = -0.049, -0.018), hearing impairment (β = -0.070, 95% CI = -0.086, -0.055), and dual sensory impairment (β = -0.054, 95% CI = -0.070, -0.039); and cognition was negatively associated with vision impairment (β = -0.028, 95% CI = -0.044, -0.013), hearing impairment (β = -0.074, 95% CI = -0.090, -0.059), and dual sensory impairment (β = -0.052, 95% CI = -0.067, -0.036), even after adjusting for demographics, social economic factors, and lifestyle behavior.

**Conclusions:**

Vision and hearing impairment are negatively associated with memory, mental status, and cognition for middle-aged and elderly Chinese adults. There were stronger negative associations between sensory impairment and cognitive-related indicators in the elderly compared to the middle-aged.

Cognitive impairment has been defined as a decline in the ability to remember, learn new things, and concentrate, which affects a person’s daily life [1]. It is estimated that by 2050, China’s dementia population will reach 10 million, which will create an overwhelming burden for individuals, families, and health care systems [2,3]. Thus, understanding the relevant factors of cognitive impairment is necessary to ameliorate cognitive impairment and prevent further loss.

Recent evidence has demonstrated that sensory impairment, including hearing or vision impairment, is associated with a higher risk of cognitive impairment. About 94% of the elderly with cognitive impairment have hearing loss [4], and about 32.5% of dementia patients have vision loss [5]. Previous studies have found that listeners with hearing loss showed lower accuracy, increased reaction times, and reduced sensitivity compared to normal hearing listeners in vocal emotion recognition [6]. In a longitudinal study, sensorineural impairment in vision was associated with the incidence of cognitive impairment in middle-aged adults [7]. According to the baseline examination of the Beaver Dam Offspring Study, hearing and visual impairment were associated with poorer performance in cognitive functions independent of the other sensory impairments and factors associated with cognition in middle-aged adults [8]. Furthermore, visual function and symptoms have been identified as predictors of dementia and outcomes in Parkinson disease [9,10]. Moreover, dual sensory impairment refers to the simultaneous presence of visual impairment and hearing impairment, and its incidence is as high as 11% in adults over 80 years old [11]. Among black and white older adults, the risk of developing dementia increased in a dose–response way with increasing number of sensory impairments [12]. A long-term prospective study based on the data from the Health and Retirement Study (HRS) showed that individuals with combined hearing and visual impairments have a 38% higher risk of incident possible CIND than individuals with no sensory impairment [13]. Recent studies have shown that elderly people with sensory and cognitive impairments, as a particularly common and vulnerable population, are at increased risk of hospitalization and lead to higher health care expenditures [14]. To explore the complex relationship between sensory impairment and cognitive impairment is of great significance to better formulate medical economic policies and help reduce the burden of social pension all over the world.

Few studies have focused on the role of sensory impairment in cognitive decline among people over 45 years old throughout China. Most previous studies have shown the relationship between a single sensory impairment and cognitive function impairment regardless of possible impairments in the other sensory systems. However, the China Health and Retirement Longitudinal Study (CHARLS) baseline data set reported information from Chinese people with or without hearing impairment, vision impairment, or dual sensory impairment. The purpose of this study was to verify if hearing impairment and vision impairment are independently associated with cognitive impairment, including the impairment of memory, mental status, and cognitive function, in middle-aged and elderly people in China.

## METHODS

### Study population

The data for this study was derived from the CHARLS baseline data set, which was run by the National School for Development at Peking University (http://charls.pku.edu.cn). The CHARLS study aims to establish a high-quality open database that includes information from a wide range of socioeconomic conditions and personal health conditions to facilitate research on the middle-aged and elderly. The participants include members aged 45 years and older from randomly selected households in 150 counties or districts and 450 villages or urban areas distributed across 28 provinces, generally representing the middle-aged and elderly people in China. The national baseline survey for CHARLS was conducted between June 2011 and March 2012 and included 17 708 respondents [15].

### Assessment of sensory impairment

Sensory impairment was assessed by asking, “Do you have vision or/and hearing problems?” We categorized sensory impairment into four groups: no sensory impairment, vision impairment, hearing impairment, and dual sensory impairment, comprising vision and hearing impairment.

### Assessment of cognition

Based on the American Health and Retirement Study, CHARLS employed items from the Telephone Interview of Cognitive Status (TICS) battery [16]. Two factors were included to measure cognitive function: memory and mental status. Memory was evaluated by immediate and delayed word recall. The subjects heard 10 unrelated Chinese words and were asked to recall them immediately (immediate word recall) and then four minutes later (delayed word recall). Mental status was assessed from three dimensions, including orientation, visuoconstruction, and mathematical performance. Orientation measures included naming today’s date (day, month, and year), the day of the week, and the season. Visuoconstruction asked participants to repaint pictures presented to them. Additionally, serial subtraction of 7 from 100 (up to five times) was used to evaluate the subjects’ mathematical performance. The above items were combined to evaluate the participants’ cognitive function [17].

### Assessment of covariates

Using the CHARLS questionnaire, we obtained information on some covariates in the present study, including age, sex, residence (urban or rural), education (primary or below, middle school, high school, or college or above), smoking status, drinking status (none, drink but less than once a month, or drink more than once a month), hypertension, diabetes, and dyslipidemia. Age was categorized as middle aged (≥45 and <60 years) and elderly (≥60 years). Hypertension was defined as mean systolic blood pressure ≥140 mm Hg and/or mean diastolic blood pressure ≥90 mm Hg, and/or self-reported use of antihypertensive medicine within two weeks according to the 2010 Chinese guidelines for hypertension [18]. The definition of diabetes comprised a subject having fasting blood glucose levels ≥7.0 mmol/L or currently taking hypoglycemic medicine. The diagnosis of dyslipidemia refers to total cholesterol ≥240 mg/dl, high-density lipoprotein cholesterol <40 mg/dl, low-density lipoprotein cholesterol >160 mg/dl, or triglycerides ≥200 mg/dl based on the 2016 Chinese guidelines for dyslipidemia in adults [19].

### Statistical analysis

Continuous variables were presented as mean ± standard deviation (SD), whereas categorical variables were presented as numbers and percentages. For a comparison among the different groups, one-way analysis of variance (ANOVA) or the Kruskal-Wallis test for continuous variables and the χ^2^ test for categorical variables were used to evaluate differences. Pearson correlation analysis was performed to evaluate the relationships between the covariates and cognition. To evaluate the relationship between sensory impairment and cognition, weighted multiple linear regression was used to fit three sets of models we built. Model 1 adjusted for age and sex. Model 2 adjusted for the covariates in model 1 plus marital status, residence, education, smoking status, and drinking status. Model 3 adjusted for the covariates in model 2 plus hypertension, dyslipidemia, and diabetes. Because CHARLS has a complex sampling design, sampling weights were considered in the regression analyses. To examine the robustness of the results, a sensitivity analysis was conducted according to age.

## RESULTS

### Characteristics of samples

Among the included participants, subjects aged under 45 years or with incomplete demographic or health status data were excluded. Thus, 13 914 individuals were included in the final analysis, including 6692 males (48.1%) and 7222 (51.9%) females. The baseline demographic characteristics of samples according to sensory impairment status are summarized in **Table 1**. Those who had sensory impairment were older, more likely to be rural, and possessed a lower level of education. Moreover, people with sensory impairments were more likely to be smokers and have hypertension and diabetes. Interestingly, sensory impairments were accompanied by decline in the function of memory, cognition, and mental status.

**Table 1:** Demographic characteristics of middle-aged and elderly Chinese with and without dual sensory impairment

Variables	No sensory impairment	Vision impairment	Hearing impairment	Dual sensory impairment	*P*-value
**No. subjects (%)**	12 143 (87.3)	553 (4.0)	924 (6.6)	294 (2.1)	
**Age, year**	58.12 ± 9.60	62.66 ± 10.12	66.45 ± 11.29	67.70 ± 11.40	<0.001
**Sex, n (%):**					0.005
Male	5826 (48.0)	243 (43.9)	488 (52.8)	135 (45.9)	
Female	6317 (52.0)	310 (56.1)	436 (47.2)	159 (54.1)	
**Residence, n (%):**					<0.001
Urban	5110 (42.1)	169 (30.6)	295 (31.9)	79 (26.9)	
Rural	7033 (57.9)	384 (69.4)	629 (68.1)	215 (73.1)	
**Education, n (%):**					<0.001
Primary or below	7727 (63.6)	456 (82.5)	761 (82.4)	252 (85.7)	
Middle school	2708 (22.3)	69 (12.5)	97 (10.5)	27 (9.2)	
High school	1363 (11.2)	24 (4.3)	52 (5.6)	10 (3.4)	
College or above	345 (2.9)	4 (0.7)	14 (1.5)	5 (1.7)	
**Smoking status, n (%):**					0.001
Yes	4734 (39.0)	220 (39.8)	424 (45.9)	122 (41.5)	
No	7409 (61.0)	333 (60.2)	500 (54.1)	172 (58.5)	
**Drinking status, n (%):**					0.246
Drink more than once a month	3079 (25.3)	128 (23.1)	213 (23.0)	69 (23.5)	
Drink but less than once a month	979 (8.1)	38 (6.9)	75 (8.1)	17 (5.8)	
None	8085 (66.6)	387 (70.0)	636 (68.9)	208 (70.7)	
**Hypertension, n (%):**					<0.001
Yes	2879 (23.7)	168 (30.4)	257 (27.8)	96 (32.7)	
No	9264 (76.3)	385 (69.6)	667 (72.2)	198 (67.3)	
**Diabetes, n (%):**					<0.001
Yes	664 (5.5)	59 (10.7)	56 (6.1)	26 (8.9)	
No	11 479 (94.5)	494 (89.3)	868 (93.9)	268 (91.1)	
**Dyslipidemia, n (%):**					0.727
Yes	1097 (9.0)	50 (9.0)	87 (9.4)	32 (10.9)	
No	11 046 (91.0)	503 (91.0)	837 (90.6)	262 (89.1)	
**Memory**	3.41 ± 2.22	2.94 ± 1.97	2.42 ± 2.12	2.36 ± 1.96	<0.001
**Mental status**	6.74 ± 3.60	5.46 ± 3.43	4.99 ± 3.72	4.44 ± 3.61	<0.001
**Cognition**	10.15 ± 5.17	8.41 ± 4.73	7.41 ± 5.32	6.81 ± 5.01	<0.001

### The correlation between cognition and covariates

The correlations between cognition and the covariates were analyzed by Pearson correlation analysis and are displayed by correlation coefficients and their *P*-values in **Table 2**. Significant correlations were found between cognition and independent variables, such as age, residence, education, and sensory impairment. Significant and negative correlations were found between age and memory (*r* = -0.222, *P* < 0.001), mental status (*r* = -0.196, *P* < 0.001), and cognition (*r* = -0.231, *P* < 0.001), which implies that memory, mental status, and cognitive function decrease with the increase of age. The associations between the level of education and memory (*r* = 0.246, *P* < 0.001), mental status (*r* = 0.307, *P* < 0.001), and cognition (*r* = 0.319, *P* < 0.001) were significant and positive with the correlation coefficient estimated as 0.246 (*P* < 0.001), 0.307 (*P* < 0.001), and 0.319 (*P* < 0.001), respectively. Similar significant tendencies were found for the correlations between residence and memory (*r* = 0.119, *P* < 0.001), mental status (*r* = 0.190, *P* < 0.001), and cognition (*r* = 0.183, *P* < 0.001). Significant and negative correlations were also found between sensory impairment and memory (*r* = -0.131, *P* < 0.001), mental status (*r* = -0.156, *P* < 0.001), and cognition (*r* = -0.164, *P* < 0.001), which indicates that memory, mental status, and cognitive function decreased with the increase of sensory impairment.

**Table 2:** Correlation between covariates and cognition

	Memory		Mental status		Cognition	
**Variables**	***r***	***P*-value**	***r***	***P*-value**	***r***	***P*-value**
Age	-0.222	<0.001	-0.196	<0.001	-0.231	<0.001
Sex	0.016	0.055	0.156	<0.001	0.115	<0.001
Residence	0.119	<0.001	0.190	<0.001	0.183	<0.001
Education	0.246	<0.001	0.307	<0.001	0.319	<0.001
Smoking status	0.011	0.212	0.089	<0.001	0.067	<0.001
Drinking status	-0.029	0.001	-0.078	<0.001	-0.067	<0.001
Hypertension	-0.017	0.047	-0.012	0.141	-0.016	<0.001
Diabetes	0.023	0.006	0.026	0.002	0.028	0.001
Dyslipidemia	0.065	<0.001	0.062	<0.001	0.071	<0.001
Sensory impairment	-0.131	<0.001	-0.156	<0.001	-0.164	<0.001

### Associations between sensory impairment and cognitive function

To further clarify the association between sensory impairment and cognitive function, we reanalyzed their relevance by controlling for the covariates in the base model (**Table 3**). Memory was negatively associated with vision impairment (β = -0.023, 95% CI = -0.039, -0.007), hearing impairment (β = -0.071, 95% CI = -0.087, -0.054), and dual sensory impairment (β = -0.040, 95% CI = -0.057, -0.054) after adjusting for age and sex. Surprisingly, memory was still negatively associated with hearing impairment (β = -0.059, 95% CI = -0.075, -0.043) and dual sensory impairment (β = -0.032, 95% CI = -0.048, -0.016) after further controlling for marital status, residence, education, smoking status, and drinking status. After further controlling for hypertension, dyslipidemia, and diabetes, memory still had significant negative associations with hearing impairment (β = -0.043, 95% CI = -0.076, -0.043) and dual sensory impairment (β = -0.033, 95% CI = -0.049, -0.017).

**Table 3:** Associations between sensory impairment and cognitive function in middle-aged and elderly Chinese*

Variables	Sensory impairment			
**No sensory impairment**	**Vision impairment**	**Hearing impairment**	**Dual sensory impairment**
**Memory:**				
Model 1	Ref	-0.023 (-0.039, -0.007)	-0.071 (-0.087, -0.054)	-0.040 (-0.057, -0.054)
Model 2	Ref	-0.011 (-0.027, 0.005)	-0.059 (-0.075, -0.043)	-0.032 (-0.048, -0.016)
Model 3	Ref	-0.011 (-0.027, 0.005)	-0.043 (-0.076, -0.043)	-0.033 (-0.049, -0.017)
**Mental status:**				
Model 1	Ref	-0.051 (-0.067, - 0.034)	-0.087 (-0.104, -0.071)	-0.066 (-0.082, -0.050)
Model 2	Ref	-0.033 (-0.048, -0.018)	-0.070 (-0.086, -0.054)	-0.054 (-0.069, -0.038)
Model 3	Ref	-0.034 (-0.049, - 0.018)	-0.070 (-0.086, -0.055)	-0.054 (-0.070, -0.039)
**Cognition:**				
Model 1	Ref	-0.045 (-0.061, - 0.029)	-0.091 (-0.107, -0.075)	-0.063 (-0.079, -0.047)
Model 2	Ref	-0.028 (-0.043, -0.012)	-0.074 (-0.090, -0.058)	-0.051 (-0.067, -0.036)
Model 3	Ref	-0.028 (-0.044, - 0.013)	-0.074 (-0.090, -0.059)	-0.052 (-0.067, -0.036)

*Model 1 adjusted for age and sex. Model 2 adjusted for covariates in model 1 plus marital status, residence, education, smoking status, and drinking status. Model 3 adjusted for covariates in model 2 plus, hypertension, dyslipidemia, and diabetes.

Furthermore, mental status was negatively associated with vision impairment (β = -0.051, 95% CI = -0.067, -0.034), hearing impairment (β = -0.087, 95% CI = -0.104, -0.071), and dual sensory impairment (β = -0.066, 95% CI = -0.082, -0.050) after adjusting for age and sex. When additionally adjusting for social economic factors and lifestyle behavior, mental status was still negatively associated with vision impairment (β = -0.033, 95% CI = -0.048, -0.018), hearing impairment (β = -0.070, 95% CI = -0.086, -0.054), and dual sensory impairment (β = -0.054, 95% CI = -0.069, -0.038). Consistently, mental status had negative associations with vision impairment (β = -0.034, 95% CI = -0.049, -0.018), hearing impairment (β = -0.070, 95% CI = -0.086, -0.055), and dual sensory impairment (β = -0.054, 95% CI = -0.070, -0.039) when controlling for hypertension, dyslipidemia, and diabetes.

Reviewing cognitive function, cognition was associated with lower visual impairment, lower hearing impairment, and lower dual sensory impairment regardless of adjusting the covariates. Cognition was negatively associated with vision impairment (β = -0.045, 95% CI = -0.061, -0.029), hearing impairment (β = -0.091, 95% CI = -0.107, -0.075), and dual sensory impairment (β = -0.063, 95% CI = -0.079, -0.047) after adjusting for age and sex. Significant negative relationships were observed between cognition and vision impairment (β = -0.028, 95% CI = -0.043, -0.012), hearing impairment (β = -0.074, 95% CI = -0.090, -0.058), and dual sensory impairment (β = -0.051, 95% CI = -0.067, -0.036) after adjusting for social economic factors and lifestyle behavior. Even if metabolism-related factors were further adjusted, there were still negative associations between cognition and vision impairment (β = -0.028, 95% CI = -0.044, -0.013), hearing impairment (β = -0.074, 95% CI = -0.090, -0.059), and dual sensory impairment (β = -0.052, 95% CI = -0.067, -0.036).

To further clarify associations between sensory impairment and cognitive function in different age groups, age was categorized into two groups: middle aged (≥45 and <60 years old) and elderly (≥60 years old; **Table 4**). In the elderly group, the multivariate-adjusted association between hearing impairment (β = -0.072, 95% CI = -0.095, -0.048) and dual impairment (β = -0.040, 95% CI = -0.064, -0.017) and decline in memory persisted significantly compared to the reference group of no sensory impairment. A significant association was observed among subjects ≥60 years old between vision impairment (β = -0.043, 95% CI = -0.065, - 0.021), hearing impairment (β = -0.090, 95% CI = -0.112, -0.067), and dual impairment (β = -0.062, 95% CI = -0.084, -0.040) and negative mental status. Vision impairment (β = -0.033, 95% CI = -0.055, - 0.011), hearing impairment (β = -0.093, 95% CI = -0.116, -0.071), and dual impairment (β = -0.061, 95% CI = -0.083, -0.039) were independently associated with decreased cognition for those ≥60 years old. In the middle-aged group, memory was negatively associated with hearing impairment (β = -0.033, 95% CI = -0.055, -0.011) and dual impairment (β = -0.010, 95% CI = -0.032, -0.012). A negative association was found between mental status and vision impairment (β = -0.019, 95% CI = -0.040, - 0.002), hearing impairment (β = -0.032, 95% CI = -0.053, -0.011), and dual impairment (β = -0.029, 95% CI = -0.050, -0.008) among middle-aged people. There was also a significant association between vision impairment (β = -0.019, 95% CI = -0.040, - 0.002), hearing impairment (β = -0.037, 95% CI = -0.058, -0.016), and dual impairment (β = -0.025, 95% CI = -0.046, -0.004) and a decline of cognition among middle-aged individuals.

**Table 4:** Associations between sensory impairment and cognitive function in middle-aged and elderly Chinese among age group*

Variables	Sensory impairment			
**No sensory impairment**	**Vision impairment**	**Hearing impairment**	**Dual sensory impairment**
**≥60 y:**				
**Memory:**				
Model 1	Ref	-0.020 (-0.044, 0.004)	-0.088 (-0.112, -0.063)	-0.052 (-0.077, -0.028)
Model 2	Ref	-0.004 (-0.027, 0.019)	-0.071 (-0.095, -0.047)	-0.039 (-0.062, -0.016)
Model 3	Ref	-0.005 (-0.029, 0.018)	-0.072 (-0.095, -0.048)	-0.040 (-0.064, -0.017)
**Mental status:**				
Model 1	Ref	-0.061 (-0.084, - 0.038)	-0.112 (-0.135, -0.088)	-0.079 (-0.103, -0.056)
Model 2	Ref	-0.041 (-0.064, -0.019)	-0.089 (-0.112, -0.067)	-0.061 (-0.083, -0.039)
Model 3	Ref	-0.043 (-0.065, - 0.021)	-0.090 (-0.112, -0.067)	-0.062 (-0.084, -0.040)
**Cognition:**				
Model 1	Ref	-0.051 (-0.075, - 0.028)	-0.115 (-0.139, -0.092)	-0.078 (-0.101, -0.054)
Model 2	Ref	-0.031 (-0.053, -0.009)	-0.093 (-0.115, -0.070)	-0.059 (-0.081, -0.037)
Model 3	Ref	-0.033 (-0.055, - 0.011)	-0.093 (-0.116, -0.071)	-0.061 (-0.083, -0.039)
**≥45 and <60 y:**				
**Memory**				
Model 1	Ref	-0.025 (-0.047, -0.003)	-0.045 (-0.067, -0.023)	-0.018 (-0.040, 0.05)
Model 2	Ref	-0.013 (-0.034, 0.009)	-0.033 (-0.054, -0.011)	-0.010 (-0.032, 0.016)
Model 3	Ref	-0.013 (-0.034, 0.009)	-0.033 (-0.055, -0.011)	-0.010 (-0.032, -0.012)
**Mental status**				
Model 1	Ref	-0.038 (-0.060, - 0.016)	-0.051 (-0.073, -0.029)	-0.041 (-0.063, -0.019)
Model 2	Ref	-0.019 (-0.040, -0.002)	-0.032 (-0.053, -0.011)	-0.029 (-0.051, -0.008)
Model 3	Ref	-0.019 (-0.040, - 0.002)	-0.032 (-0.053, -0.011)	-0.029 (-0.050, -0.008)
**Cognition**				
Model 1	Ref	-0.037 (-0.060, - 0.015)	-0.055 (-0.077, -0.033)	-0.036 (-0.058, -0.014)
Model 2	Ref	-0.019 (-0.040, -0.002)	-0.036 (-0.057, -0.015)	-0.025 (-0.046, -0.004)
Model 3	Ref	-0.019 (-0.040, - 0.002)	-0.037 (-0.058, -0.016)	-0.025 (-0.046, -0.004)

*Model 1 adjusted for age and sex. Model 2 adjusted for covariates in model 1 plus marital status, residence, education, smoking status, and drinking status. Model 3 adjusted for covariates in model 2 plus, hypertension, dyslipidemia, and diabetes.

## DISCUSSION

With the aging of the global population, the increasing number of people suffering from cognitive dysfunction, doubling every 20 years, has become an imminent public crisis [20]. Most dementia patients are cared for by family members in the community, which bring their family caregivers great mental and physical pressure [21]. Thus, it is very important to explore and intervene factors related to cognitive function.

In the present study, we first analyzed and confirmed the negative association between hearing, vision, and dual sensory impairment and cognition impairment across middle-aged and elderly Chinese adults. Further adjustments for demographics, social economic factors, and lifestyle behavior lowered the association slightly, but it remained significant.

Memory, mental status, and cognitive function declined with the increase of age, drinking, and smoking status, which is consistent with previous studies [22-24]. However, these three cognitive-related indicators were positively associated with education and dyslipidemia. In previous studies evaluating dyslipidemia and cognitive functions, the findings were inconsistent, and mixed results were reported [25-27]. A recent study among 90 multiple sclerosis inpatients reported that a high level of triglycerides could be associated with an increased risk of cognitive impairment [25]. One possible reason for this is that while CHARLS participants are generalized to all middle-aged and older Chinese adults, this small study focused on multiple sclerosis patients. However, a 21-year follow-up study showed that a high midlife TC level comprised a risk factor for cognitive dysfunction in later life, whereas a low TC level was significantly associated with the risk of cognitive impairment late in life [27], which is consistent with our present study.

Additionally, sensory impairment was significantly associated with worse memory, mental status, and cognition. Vision impairment was negatively associated with memory, but after adjusting for marital status, residence, education, smoking status, and drinking status, the association was not significant. However, visual impairment was negatively associated with mental status and cognition, even after controlling for demographics, social economic factors, lifestyle behavior, and metabolic measures. In a Korean study of adults who were 19 years of age or older, visually impaired people showed increased risk for poor mental status [28]. Furthermore, compared to healthy volunteers, glaucoma patients have worse mental states and are more likely to be associated with anxiety or depression; in glaucoma patients, the worse vision becomes, the worse mental status becomes [29]. These analyses support the results in our present study, specifically, the negative association between visual impairment and mental status. Similar to our findings, a NHANES and NHATS study reported that vision dysfunction was associated with poor cognitive function [30]. Recent studies have further suggested that there may be a causal relationship between worsening vision and future declining cognition [31]. As a result, retinal measurements of spectral-domain OCT were reported as one of the biomarkers of cognitive decline [32]. Although several studies have investigated the association between vision impairment and mental status and cognitive function, the influence of different age groups in the middle-aged and elderly is rarely mentioned, and extensive research using a large sample like CHARLS in China is not available. The present report found that there was a stronger negative association between mental status as well as cognition and vision impairment in the elderly compared to middle-aged people. Our study contributed further knowledge that visual impairment in the elderly has a greater association with cognition and mental status, which might impose a burden on normal daily life. Therefore, preventing, reducing, and correcting vision disorders early might lead to a decrease in cognition impairment among middle-aged and older adults.

Furthermore, hearing impairment was negatively associated with memory, mental status, and cognition, even after adjusting for age, sex, marital status, residence, education, smoking status, drinking status, hypertension, dyslipidemia, and diabetes. It is estimated that hearing loss, an independent risk factor for dementia, accounts for 9% of cases in middle-aged people [33]. There are some putative biological mechanisms for the relationship between hearing loss and dementia [34-36]. First, the pathology of the cerebral cortex that causes dementia also appears in the acoustic environment, including the cochlea and ascending auditory pathway [37-40]. Second, hearing loss causes an impoverished environment and negatively affects brain structure and function, leading to decreased cognitive reserves and decreased resilience to dementia [41-45]. Moreover, hearing impaired people utilize a portion of their cognitive resources to listen, which may result in the diminished availability of cognitive resources in the brain [35,46-48]. The occupied theory might also be a more agreeable explanation for age-related hearing loss. Additionally, hearing loss alters cortical activity, and there is an interaction between this altered activity and Alzheimer disease pathology, such as neurofibrillary changes associated with Tau pathology [36,49-51]. In our present study, there were stronger negative associations between hearing impairment and cognitive related indicators in the elderly compared to the middle-aged. Therefore, early hearing intervention for hearing impaired people may improve cognitive function to a certain extent. However, the improvement of cognitive function by using only hearing aids is limited and inconsistent [52-55], which may be due to the complex mechanism of age-related hearing loss. Further studies are thereby warranted to explore the cognitive benefits of multiple hearing interventions.

In the present study, dual sensory impairment was negatively associated with memory, mental status, and cognition, even after adjusting for other confounding factors. The association between dual sensory impairment and cognition is greater than that of visual impairment alone but not as great as that of hearing impairment alone. Few prior, but inconsistent, studies have reported the impact of dual sensory impairments on memory, mental status, and cognition. Participants with dual sensory impairment, rather than Alzheimer disease, were more likely to be diagnosed with dementia, but the average age of the participants was already 82.8 years old, and they were homecare clients [56]. In a longitudinal analysis, the population with dual vision and hearing impairment showed the greatest risk for cognitive function decline with the CIs overlapped with those for single impairment alone [57]. Based on existing research, it is difficult to say that dual sensory impairment confers greater risks for memory, emotional state, and cognition than hearing impairment alone.

Some limitations in our study should also be considered. First, as a cross-sectional analysis, our study cannot confirm the causal relationship between sensory impairment and cognitive impairment. Sensory impairment itself may be the result of cognitive impairment. Thus, we can only conclude that there is a significant negative association between sensory impairment and cognition. Second, the data on sensory impairment was self-reported. Although there are some differences between self-report and clinical data in evaluation, there are some studies that support the reliability of self-reported sensory disorders [58-60]. Therefore, the results involved in the study are still of great significance.

## CONCLUSIONS

In conclusion, the current study provides additional evidence that vision and hearing impairment are negatively associated with memory, mental status, and cognition for middle-aged and elderly Chinese adults. The results suggest that early interventions for vision and hearing disorders may improve the cognitive function of middle-aged and elderly people. Further research is needed to confirm our findings and help elderly people improve cognition.
